# A multi-criteria spatial deprivation index to support health inequality analyses

**DOI:** 10.1186/s12942-015-0004-x

**Published:** 2015-03-20

**Authors:** Pablo Cabrera-Barona, Thomas Murphy, Stefan Kienberger, Thomas Blaschke

**Affiliations:** Interfaculty Department of Geoinformatics - Z_GIS, University of Salzburg, Schillerstraße 30, 5020 Salzburg, Austria

**Keywords:** Deprivation, Analytical Hierarchy Process (AHP), Ordered Weighted Averaging (OWA), Geographically Weighted Regression (GWR), Health, Privación, Proceso Analítico Jerárquico, Sumatoria Lineal Ordenada Ponderada, Regresión Ponderada Geográficamente, Salud

## Abstract

**Background:**

Deprivation indices are useful measures to analyze health inequalities. There are several methods to construct these indices, however, few studies have used Geographic Information Systems (GIS) and Multi-Criteria methods to construct a deprivation index. Therefore, this study applies Multi-Criteria Evaluation to calculate weights for the indicators that make up the deprivation index and a GIS-based fuzzy approach to create different scenarios of this index is also implemented.

**Methods:**

The Analytical Hierarchy Process (AHP) is used to obtain the weights for the indicators of the index. The Ordered Weighted Averaging (OWA) method using linguistic quantifiers is applied in order to create different deprivation scenarios. Geographically Weighted Regression (GWR) and a Moran’s I analysis are employed to explore spatial relationships between the different deprivation measures and two health factors: the distance to health services and the percentage of people that have never had a live birth. This last indicator was considered as the dependent variable in the GWR. The case study is Quito City, in Ecuador.

**Results:**

The AHP-based deprivation index show medium and high levels of deprivation (0,511 to 1,000) in specific zones of the study area, even though most of the study area has low values of deprivation. OWA results show deprivation scenarios that can be evaluated considering the different attitudes of decision makers. GWR results indicate that the deprivation index and its OWA scenarios can be considered as local estimators for health related phenomena. Moran’s I calculations demonstrate that several deprivation scenarios, in combination with the ‘distance to health services’ factor, could be explanatory variables to predict the percentage of people that have never had a live birth.

**Conclusions:**

The AHP-based deprivation index and the OWA deprivation scenarios developed in this study are Multi-Criteria instruments that can support the identification of highly deprived zones and can support health inequalities analysis in combination with different health factors. The methodology described in this study can be applied in other regions of the world to develop spatial deprivation indices based on Multi-Criteria analysis.

## Resumen

**Antecedentes:**

Índices de privación son medidas útiles para analizar inequidades en salud. Existen varios métodos para construir estos índices, sin embargo pocos estudios han usado Sistemas de Información Geográfica (SIG) y métodos Multi-Criterio para esta construcción. Este estudio aplica Evaluación Multi-Criterio para calcular los pesos de los indicadores del índice de privación, y también un enfoque SIG de lógica difusa para crear distintos escenarios de este índice.

**Métodos:**

El Proceso Analítico Jerárquico (AHP) es usado para obtener los pesos de los indicadores del índice. La Sumatoria Lineal Ordenada Ponderada (OWA) que usa cuantificadores lingüísticos es aplicada para crear diferentes escenarios de privación. La Regresión Ponderada Geográficamente (GWR) y el índice Moran’s I son empleados para explorar relaciones espaciales del índice de privación y sus escenarios, con dos factores relacionados a salud: distancia a servicios de salud y porcentaje de personas que nunca han tenido un nacido vivo. Este último indicador fue considerado como la variable dependiente de la GWR. El caso de estudio es la Ciudad de Quito, en Ecuador.

**Resultados:**

El índice basado en el método AHP muestra media y alta privación (0,511 a 1,000) en zonas específicas del área de estudio, no obstante, la mayoría del área de estudio tiene bajos niveles de privación. Los resultados de OWA muestran escenarios de privación que pueden ser evaluados considerando diferentes actitudes de los tomadores de decisión. Los resultados de GWR indican que el índice de privación y sus escenarios OWA pueden ser considerados como estimadores locales de fenómenos relacionados a la salud. Los cálculos de Moran’s I demuestran que varios escenarios de privación, en combinación con el factor de ‘distancia a servicios de salud’, podrían ser variables explicativas del porcentaje de personas que nunca han tenido un nacido vivo.

**Conclusiones:**

El índice basado en el método AHP y los escenarios OWA de privación son instrumentos de análisis Multi-Criterio que pueden apoyar a la identificación de zonas con pobreza, y en combinación con otros factores de salud, pueden apoyar al análisis de inequidades en salud. La metodología descrita puede ser aplicada en otras regiones del mundo para desarrollar índices de privación basados en análisis Multi-Criterio.

## Background

Approaches to developing deprivation indices are diverse [1-4], and area-based deprivation indices have been proven to be useful in identifying patterns of inequalities in health outcomes [1-11]. Deprivation can be defined as any disadvantage of an individual or human group, related to the community or society to which the individual or human group belongs, and these disadvantages can be of social or material nature [4,5]. Social deprivation can be linked to concepts of social fragmentation [11], and material deprivation can be related to the concept of poverty in terms of the lack of basic goods. These two kinds of deprivation are closely linked to public health and wellbeing [12]. Measuring deprivation requires the identification of two main issues: which indicators to be used to construct a deprivation index, and how to combine these indicators. The criteria for choosing the different indicators that compose deprivation indices can vary. In general, they depend on the availability of information in census and the objective of the study [2-4,8,9]. There are referential studies on constructing multiple deprivation indices, such as the Townsend Deprivation Index, which uses four indicators of material and social deprivation [4]; the Under Privileged Area score, also known as the Jarman Deprivation score, which considers eight deprivation indicators, and has been used to determine remuneration for physicians in United Kingdom [13,14]. Another known measure is the Carstairs deprivation index [15], which is very similar to the Townsend index but is a Scottish reality-based index. Common indicators for these three indices are overcrowding and unemployment. Townsend and Carstairs indices also include a very specific variable available in the British Census, namely the indicator of “Non car ownership”. More recent efforts have used other kinds of indicators from different domains, including health, housing and vulnerability of the population, for the construction of deprivation indices [1-3,6-9]. However, the most common deprivation domains that can support studies of health are related to occupation, education and household conditions, including overcrowding [3]. Once the indicators for a deprivation index are chosen, the next important step is to define how they are going to be combined. Deprivation indicators can be combined using (i) simple additive techniques, using (ii) weights for each indicator, or using (iii) multivariate techniques [16]. The first technique just adds the deprivation indicators [4,16], the second technique can include expert-based weights [17], and the third technique commonly uses indicators weights created using statistical analysis such as the Principal Component Analysis [2,18].

Deprivation indices are constructed by integrating indicators generally extracted from census areas data [18,19]. In many parts of the world where census data are available, such indices can be geo-referenced using GIS. Subsequently, such geo-referenced data allow further spatial analyses, such as investigating spatial correlations [20], performing accessibility analysis [21], analyzing geographical patterns [22] or studying multiple scale evaluations [10] of deprivation measures.

However, regarding the capacity of deprivation indices to be represented spatially explicit, there has been surprisingly little discussion so far about the spatial perspectives of these indices [10,17,22]. There is also not much documented experience - at least not through systematic comparisons of different scenarios - on how to construct these indices spatially.

Based on this background, this paper shows the development of a deprivation index using techniques from Multi-Criteria decision making [8,17,23] and GIS-based fuzzy methods [17,24]. This methodology will show how an Analytical Hierarchy Process (AHP) is applied to obtain the weights for the different indicators that make up the deprivation index. AHP is a Multi-Criteria evaluation method that takes information from experts’ judgments [23]. We then apply Ordered Weighted Averaging (OWA) in order to create different deprivation scenarios [17]. The indicators used to construct our spatial deprivation index follow a rights-based approach [25,26], and are extracted from the 2010 Ecuadorian Population and Housing Census. This rights-based approach prioritizes latent problems in Latin America, where basic needs problems (for example, not having sewerage systems) are more common than, for example, in European countries. The indicators used represent education, health, employment and housing conditions in census blocks of our study area, Quito City, Ecuador. This area has a total of 4034 census blocks, and the census block is considered the smallest area from which census information could be extracted.

A spatial explorative analysis using Geographically Weighted Regression (GWR) and Moran’s I is applied to the deprivation index and its scenarios to evaluate how they are spatially related to the following health factors: distance to health services, and the percentage of people that have never had a live birth. The distance to health services is considered a variable of health accessibility that could be considered in relation to deprivation measures in order to identify its effects on health [21]. The health factor of the percentage of people that have never had a live birth is related to a Population Census variable called “number of people that have never had a live birth”. This indicator can represent health inequalities: when a woman’s child is not born alive, this could be considered to be a consequence of a health condition, such as reproductive or maternal health problem [27]. This indicator can be calculated using information available in the 2010 Ecuadorian Population and Housing Census, and therefore could be considered a useful health-related indicator that can be analyzed together with deprivation indices to be obtained from future Census data. This variable is obtained from women’s answers about how many live births they have had. At the time of a child’s birth, he or she is considered to be a “live birth” if he or she shows vital life signals such as breathing and movement.

## Methods

### Study area and materials

**Figure 1:**
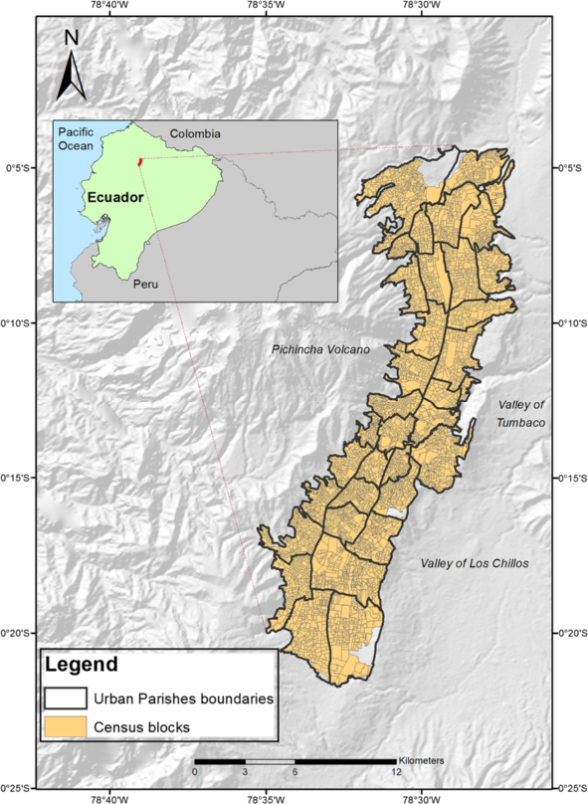
**Location of the case study.**

The case study is the urban area of the Metropolitan District of Quito, Ecuador (Figure 1). This area is known as Quito City, and is home to more than 1.5 million people distributed in 34 urban districts (Parishes) [28]. This urban area has a narrow shape due its limits with the Pichincha Volcano in the west and the Valleys of Tumbaco and Los Chillos to the east. Over 80% of inhabitants are *mestizos* (mixed-ethnicity people) [28] but the city is also inhabited by minorities such as indigenous people, black people and white people. Historically, the south of Quito City was home to blue collar workers, as well as being the area where several factories and companies have settled [29]. In contrast, the north was inhabited by wealthier people. However, due the influx of migrants from other areas of the country and the population growth [30], there is not a single rule to locate different socio-economic groups in the city today, and we can find very poor neighborhoods in the north, and very new and up-market condominiums in the south.

The information to construct the deprivation index was derived from the 2010 Ecuadorian Population and Housing Census [28]. The advantages of using Population and Housing Census information to construct indices are that census data are commonly open access, and follow a standardization that allows a comparison of information between different places and time. A geocoded shape file of census blocks was also used in order to link the 2010 Census data to the 4034 census areas that make up the study area.

For the calculation of the distance to health services, a data set of the geo-referenced health services in Quito City was used. This data set was provided by Ecuador’s Ministry of Health.

### Multi-Criteria Evaluation

A Multi-Criteria Evaluation (MCE) includes knowledge derived from different resources that can be integrated with GIS methods in order to support different kinds of analyses [23]. MCE combines information obtained from different criteria to produce an evaluation index [31] and a weight is allocated to each criterion, to represent the importance of the criterion. In this study, the Analytical Hierarchy Process (AHP) was applied. AHP is a MCE method developed by Saaty [32] that offers practical support for decision making and a straightforward way to obtain weights from criteria [23]. MCE methods, including AHP, also have the capacity to be integrated into GIS-based environments [23,24,33-36] and these GIS-based MCE approaches have been widely and successfully applied in environmental analysis [23,24,31,33,35,37].

The first step in an AHP is to identify a set of criteria. For the deprivation index developed in this study, the criteria are factors or variables that are considered to determine deprivation. A rights-based approach was used to choose the factors that make up the deprivation index [25,26], taking into account the framework of *Buen Vivir* (Good Living), that is based on human rights and nature rights. The *Buen Vivir* concept considers that in order to achieve a better quality of life, including time for leisure and harmony with nature, basic needs should first be satisfied [26]. *Buen Vivir* cannot be achieved if people do not have access to services that ensure their wellbeing and allow them to develop capabilities that create equal opportunities for everyone [26]. To have a good education, health, and to live in conditions of dignity, encourages actions that allow people to construct cohesive societies of Good Living. Human rights are universal. Therefore, the *Buen Vivir* concept can be applied in other countries, and it is not a concept which focuses only on Ecuador.

Table 1 shows the different indicators considered for the construction of the deprivation index. Each indicator is considered as a criterion for the AHP. The chosen indicators fulfill the following requirements: i) to consider a human rights-based approach, ii) to be related to health and to have an affinity with material or social dimensions of deprivation [2,3,11,18,21] and iii) to be able to be represented at the census block level [2]. The chosen indicators belong to the dimensions of education, health, employment and housing conditions.

**Table 1:** **Criteria to construct the deprivation index**

**Dimension**	**Variable**	**Indicator**/**Criteria**
Education	Level of instruction	**A**: % of people without any level of instruction
Health	Health insurance	**B**: % of people with no health insurance
	Disabled people	**C**: % of people that area disable for more than a year
Employment	Workers with no payment	**D**: % of people that work with no payment
Housing	House structure	**E**: % of houses with damaged roofs
	Overcrowding	**F**: % of houses with more than four people per dormitory
	House services	**G**: % of houses without sewerage system
		**H**: % of houses without connection to the electric grid
		**I**: % of houses without connection to the drinking water supply system
		**J**: % of houses without cobbled or paved access roads
		**K**: % of houses without garbage collection service
	House general condition	**L**: % of houses that are shacks

The indicators used to construct the index represent socio-economic problems: people with no education and people that work for no payment. People who are physically disabled for over a year will be limited in their normal work and daily activities, and those without insurance will be extremely disadvantaged when it comes to health care services. The housing indicators used represent limitations of access to services and a lack of quality of life in the households.

Variances Inflation Factors (VIF) were calculated for all indicators used in order to identify multi-colinearities. VIF shows how much the variance of an estimated regression coefficient is increased as a result of the colinearities between two variables. All VIF obtained were less than 5, which means all selected indicators can be used for the construction of the index.

The key step in any AHP is the creation of a pairwise comparison matrix to compute weights for each criterion while reducing the complexity of the phenomenon in question, because only two criteria are compared at one time [38]. For the comparison of the resulting pairwise matrix, a unified scale is used. The grade of importance of each indicator is evaluated in relation to all other indicators. The importance scale ranges from 1 to 9, whereby 1 means equal importance, 3 means moderate importance, 5 means strong or essential importance, 7 represents very strong importance and 9 indicates extreme importance. Values of 2, 4, 6 and 8 can also be used and are considered as intermediate values.

In order to obtain the references for the grades of importance, 32 experts’ judgments were taken into consideration. The consulted experts are members of public and private Ecuadorean Institutions and work in the fields of Medicine, Geography and Territorial Planning, Environmental Sciences, and Social Sciences. They were consulted via an online questionnaire in September, 2014. The results of the pairwise comparison matrix are shown in Table 2, and the importance scores show that, according to the experts, the chosen indicators are of equal or very similar importance: for example, indicator B (% of people with no health insurance) is of the same importance as indicator A (% of people without any level of instruction), and indicator D (% of people that work with no payment) is of moderately greater importance than indicator C (% of people that are disabled for more than a year). The pairwise comparison matrix is reciprocal, consequently it is only necessary to fill in one diagonal half of the matrix. After assigning the different levels of importance in the pairwise comparison matrix, a normalized matrix (*N*) is obtained as described below [39]:

$$ N = \frac{a_{ij}}{{\displaystyle \sum }{a}_{ij}} $$

**Table 2:** **Results of the AHP method**

**Indicator (Criteria)**	**A**	**B**	**C**	**D**	**E**	**F**	**G**	**H**	**I**	**J**	**K**	**L**	**Weights** ***w***_***j***_
A	1												0,0757
B	1	1											0,0757
C	1/2	1/2	1										0,0408
D	2	2	3	1									0,1410
E	1	1	2	1/2	1								0,0757
F	1	1	2	1/2	1	1							0,0757
G	2	2	3	1	2	2	1						0,1410
H	1	1	2	1/2	1	1	1/2	1					0,0757
I	2	2	3	1	2	2	1	2	1				0,1410
J	1/2	1/2	1	1/3	1/2	1/2	1/3	1/2	1/3	1			0,0408
K	1	1	2	1/2	1	1	1/2	1	1/2	2	1		0,0757
L	1/2	1/2	1	1/3	1/2	1/2	1/3	1/2	1/3	1	1/2	1	0,0408

Consistency ratio (*CR*): 0,0019.

The normalized value for each cell of *N* is obtained by calculating the ratio of each importance value *α*_*ij*_ of the pairwise comparison matrix and the values sum of each column of this matrix.

Afterwards, all the row values of the normalized matrix are added, and then the sum is divided by the number of the indicators used to construct the deprivation index. The result of this operation is a vector that contains the weights for each indicator (criterion), the eigenvector.

One of the potentials of AHP is that one can evaluate the consistency of the experts’ judgments, by calculating a consistency ratio (*CR*) that indicates the likelihood that the pairwise comparison matrix judgments were generated randomly [32]:

$$ CR=\frac{CI}{RI} $$

Were *CI* is the consistency index and *RI* is the random index. *CI* is calculated using the equation:

$$ CI=\frac{\lambda_{max}-n}{n-1} $$

Where *n* represents the number of criteria and *λ*_*max*_ is obtained as follows: a second vector is obtained by multiplying the eigenvector and the pairwise comparison matrix. Then a third vector is obtained by dividing the values of the second vector by the values of the eigenvector. *λ*_*max*_ is the average of all the components of this final vector [39].

*RI* represents the consistency index of a random pairwise comparison matrix [38] and the values that this index can take depends of the number of criteria used [39]. Table 3 shows different values for the *RI*. In this study, we worked with twelve criteria or indicators, therefore the *RI* value used is 1,48.

**Table 3:** **Random indices**

***n***	**1**	**2**	**3**	**4**	**5**	**6**	**7**	**8**	**9**	**10**	**11**	**12**	**13**	**14**	**15**
**RI**	0	0	0,58	0,90	1,12	1,24	1,32	1,41	1,45	1,49	1,51	1,48	1,56	1,57	1,59

The *CR* obtained was 0,0019, a value lees than 0,10. This value means that the pairwise comparison matrix is satisfactory [39], which is to say that there is a reasonable level of consistency in the experts’ judgments [38,40]. The weights obtained for each indicator and the *CR* are also showed in Table 2.

A first representation of the deprivation index was calculated based on the AHP weights by adding the deprivation weighted indicators. Linear min-max normalization was applied to this deprivation index. Values closer to 1 represent higher deprivation. We call the result of this calculation the AHP-based deprivation index.

### Ordered Weighted Averaging (OWA)

The Ordered Weighted Averaging (OWA) provides an extension of the Boolean and weighted aggregation operations [39,41]. It ranks the criteria in a MCE and addresses the uncertainty from criteria interaction [24]. OWA works not only with criteria weights (*w*_*j*_. j = 1,2,3,…, *n*) but principally with order weights (*v*_*j*_. j = 1,2,3,…, *n*). Criteria weights are assigned to each criterion and indicate the level of importance of each criterion [42]. We applied AHP to calculate criteria weights. On the other hand, order weights depend on the ranking of each criterion rather than on its attributes. Order weights are assigned differentially in each location, depending on the respective criterion rank order [43]:

For example, if *v*_1_, *v*_2_ and *v*_3_ are order weights that have to be applied to the AHP-based weighted criteria X, Y and Z, for instance, if at one location the rank order is YXZ and in another location it is ZYX, the order weights are assigned as *v*_1_ * Y + *v*_2_ * X + *v*_3_ * Z and *v*_1_ * Z + *v*_2_ * Y + *v*_3_ * X, respectively.

The OWA operator is defined as follows [44-46]:

$$ OW{A}_i = {\displaystyle \sum_{j=1}^n}\left(\frac{u_j{v}_j}{{\displaystyle {\sum}_{j=1}^n}{u}_j{v}_j}\right){z}_{ij} $$

Where *u*_*j*_ is the criterion weight reordered according to each criterion attribute value, *v*_*j*_ is the order weight and *Z*_*ij*_ is the sequence obtained by reordering the attribute values. When using different order weights, different results can be produced. From a GIS-based perspective, therefore, using different Boolean operations such as union (OR) and intersection (AND), or weighted linear combination [43-46] will result in different spatial patterns.

The key issue in OWA is to obtain the order weights. We used linguistic quantifies to support the production of the order weights [42,44]. Linguistic quantifiers allow to translate natural language into mathematical formulations [42]: if we consider that *Q* is a linguistic quantifier, it can be represented as a fuzzy set over the interval 0 to 1, and if we consider that *p* is a value belonging to this interval, *Q*(*p*) represents the compatibility of *p* with the concept referred to by the quantifier *Q* [42,44] and is denoted by:

$$ Q(p)={p}^{\propto },\kern0.5em \propto >0 $$

Where the parameter ∝ changes depending on which linguistic quantifier it belongs to, and can vary from, “at least one” to “all” quantifiers [38,42,44].

We used regular increasing monotone (RIM) quantifiers that produce order weights related to measures of ORness and tradeoff [42,44,46,47]. Table 4 shows the different values that the parameter ∝ can take.

**Table 4:** **Properties of Regular Increasing Monotone (RIM) quantifiers**

**∝**	**Linguistic Quantifier (Q)**	**OWA weights (** ***v***_***j***_ **)**	**Decision strategies**
→0	At least one	*v*_1_=1; *v*_*j*_=0, for all other order weights	Extremely optimistic
0,1	Few	*	Very optimistic
0,5	Some	*	Optimistic
1	Half	*v*_*j*_=1/n for all *j*	Neutral
2	Many	*	Pessimistic
10	Most	*	Very pessimistic
→∞	All	*v*_*n*_=1; *v*_*j*_=0, for all other order weights	Extremely pessimistic

*These weights are problem-specific.

In the OWA procedure, it is very important to evaluate the decision strategies. These strategies range between extremely optimistic and extremely pessimistic. These strategies are to be interpreted according to the following logic: in the extremely optimistic strategy, the decision maker’s attitude leads to weighting the highest possible outcome value (for this study the outcome value is the value of deprivation). From a probabilistic perspective, an extremely optimistic strategy is a situation in which a probability of 1, the highest probability, is assigned to the highest value at each location [45]. In other words, the highest ordered weight is assigned to the highest value at each location. The linguistic quantifier for the extremely optimistic strategy is “At least one”, and this linguistic quantifier is equivalent to the logic OR (union) [44], meaning that something is true if at least one logic operand is true.

The other extreme is the extremely pessimistic strategy, where the decision maker’s attitude leads to weighting the lowest possible outcome value. From a probabilistic perspective, in this strategy, the probability of 1 is assigned to the lowest value at each location [45]. The linguistic quantifier for the extremely pessimistic strategy is “All”, and this linguistic quantifier is equivalent to the logic AND (intersection) [44], meaning that something is true if all logic operands are true.

The neutral decision strategy represents a full-tradeoff between criteria, where equal order weights are applied to all possible values at each location. When increasing the degree of optimism from the neutral strategy, greater order weights are assigned to the higher criterion values and smaller weights to the lower criterion values.

In this study, we used the following GIS-based MCE equation to calculate order weights for OWA [44]:

$$ {v}_j={\left({\displaystyle \sum_{k=1}^j}{u}_k\right)}^{\propto }-{\left({\displaystyle \sum_{k=1}^{j-1}}{u}_k\right)}^{\propto } $$

Finally, the linguistic quantifier-based OWA is defined as follows [44]:

$$ OW{A}_i = {\displaystyle \sum_{j=1}^n}\left({\left({\displaystyle \sum_{k=1}^j}{u}_k\right)}^{\propto }-{\left({\displaystyle \sum_{k=1}^{j-1}}{u}_k\right)}^{\propto}\right){z}_{ij} $$

Table 5 provides an illustration of how to compute OWA_i_ for the case of ∝ = 2 (for the RIM equals “Many”) considering four hypothetic variables, each one with its respective weight.

**Table 5:** **Illustration of OWA calculation for four criteria values, for the linguistic quantifier 2**

**j**	**Criterion values**	**Criterion weights** ***w***_***j***_	**Ordered criterion values** ***z***_***ij***_	**Ordered criterion weights** ***u***_***ij***_	${\left({\displaystyle \sum_{\boldsymbol{k}=1}^{\boldsymbol{j}}}{\boldsymbol{u}}_{\boldsymbol{k}}\right)}^{\propto }$	***v***_***j***_	***v***_***j***_ * ***z***_***ij***_
1	0,20	0,30	0,80	0,35	(0,35)^2^ = 0,1225	(0,1225 - 0) = 0,1225	0,098
2	0,80	0,35	0,50	0,10	(0,45)^2^ = 0,2025	(0,2025 -0,1225) = 0,08	0,04
3	0,50	0,10	0,30	0,25	(0,70)^2^ = 0,49	(0,49-0,2025) = 0,2875	0,08625
4	0,30	0,25	0,20	0,30	(1)^2^ = 1	(1–0,49) = 0,51	0,102
∑		1		1		1	*OWA*_*i*_=0,33

The process described in our illustration was applied to all the 4034 census blocks of our study area, for all the 12 chosen indicators, for each one of the seven quantifiers: At least one (Extremely optimistic), Few (Very optimistic), Some (Optimistic), Half (Neutral), Many (Pessimistic), Most (Very pessimistic) and All (Extremely pessimistic).

**Figure 2:**
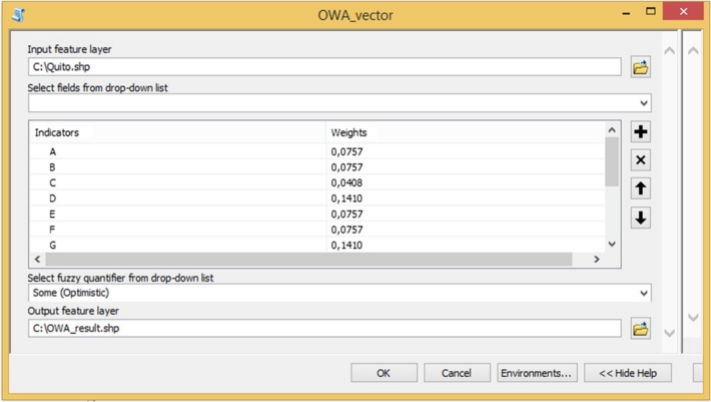
**Graphical user interface of the tool developed to compute OWA with fuzzy quantifiers.**

In order to process this large amount of information, we developed a tool to compute the Ordered Weighted Average with fuzzy quantifiers based on the method presented by Malczewski [44]. The tool is implemented as a Python toolbox in ArcGIS software (ESRI, Redlands, USA). Python is an open-source programming language that can be used in a wide variety of software application domains. Our Python toolbox uses a Python package for scientific computing called NumPy. During computation, NumPy is instructed to apply the appropriate mathematical functions to compute the Ordered Weighted Average. Using the tool requires entering a feature layer with the criteria as attributes. The Graphical User Interface of the tool is displayed in Figure 2. To use the tool, the user must browse to the feature layer, then select the criteria from the drop-down list and enter the weight for each criterion. After the criteria and the weights are entered, the fuzzy quantifier must be selected from a dropdown list. This list has seven decision strategies: At least one (Extremely optimistic), Few (Very optimistic), Some (Optimistic), Half (Neutral), Many (Pessimistic), Most (Very pessimistic) and All (Extremely pessimistic). After the decision strategy is selected, the location for the output feature layer must be entered. The output feature layer is a copy of the input feature layer with the OWA values attached as an attribute.

We applied the tool to compute the OWA for the 4034 census blocks. While the tool is running, the linguistic quantifier selected is translated to a numeric parameter ∝ where the values assigned are: 0,001 for At least one, 0,1 for Few, 0,2 for Some, 1 for Half, 2 for Many, 10 for Most and 1000 for All [44]. Using the tool, the OWA was computed for all seven decision strategies, yielding seven scenarios for the deprivation index. These seven scenarios were normalized on a scale from 0 to 1 using linear min-max normalization.

### Spatial relationships between the different deprivation measures and health factors

Two health indicators were chosen to evaluate the relation of the OWA deprivation scenarios and the AHP-based deprivation index with health: the distance of each census block to the nearest health service and the percentage of people in each census block that have never had a live birth. These two health indicators represent two different direct measures of the health dimension: a spatial measure of distances and a social measure of a health outcome. For the indicator of distance to health services, first, the centroids for each of the 4034 census blocks were calculated. Sizes of the census blocks differed all over the study area (sizes from around 3200 square meters to sizes of more than 400 000 square meters), therefore, centroids are a good representation of each census block location. Then, 128 health services were identified in the study area and the distances from each census block centroid to the nearest health service were calculated.

The indicator of percentage of people that have never had a live birth was calculated for each census block, using information available in the 2010 Ecuadorian Population and Housing Census: the “number of people that have never had a live birth” and the population of each census block.

Geographically Weighted Regression (GWR) was applied considering the measures of deprivation and distance to health services as the explanatory variables. The indicator of percentage of people that have never had a live birth was considered as the dependent variable. A different GWR was made for each OWA scenario of deprivation and for the AHP-based deprivation index. GWR is an extension of the standard regression techniques that allows parameters *β*_*k*_ to vary spatially. GWR evaluates the variations of the regression model relationships across space and, contrary to simple regressions, GWR allows local parameter estimates [48-50]. The GWR model can be written as:

$$ Y\left({s}_i\right)={\beta}_0(s)+{\displaystyle \sum_{k=1}^M}{\beta}_k(s){X}_k\left({s}_i\right)+\varepsilon \left({s}_i\right) $$

This equations means that at every location *s*, all coefficients *β*_*k*_ need to be estimated, and *ε*(*s*_*i*_) is a random error with a mean of zero and a constant variance [50].

The estimations of coefficients *β*_*k*_ require the weighting of all observations, and the weights are a function of the distance between the location *s* and the observations around this location [49]. The function to calculate the weights is the kernel function:

$$ {w}_{ij}= exp\left(\frac{h_{ij}^{\alpha }}{b}\right) $$

Where *w*_*ij*_ is the weight of location *s*_*j*_ that is used to estimate a parameter *β*_*k*_ at the location *s*_*i*_, and ${h}_{ij}^{\alpha }$ is the distance between observations *s*_*j*_ and *s*_*i*_ [50].

The aim of applying GWR in this study is to explore how the AHP-based deprivation index and its OWA scenarios relate to health factors by determining the spatial correlations of these relationships. The GWR technique is complemented with the application of the Global Moran’s I.

Moran’s I is an index to measure spatial autocorrelation by comparing the value of a variable of one location with the value of this variable at all other locations [51]. Moran’s I is defined by the following equation:

$$ I=\frac{n{\displaystyle {\sum}_{i=1}^n}{\displaystyle {\sum}_{j=1}^n}{w}_{ij}\left({x}_i-\overline{x}\right)\left({x}_j-\overline{x}\right)}{{\displaystyle {\sum}_i}{\displaystyle {\sum}_{j\ne i}}{w}_{ij}\left({\displaystyle {\sum}_{i=1}^n}{\left({x}_i-\overline{x}\right)}^2\right)} $$

Where *n* is the number of spatial units to be taken into account, *x* is a value of a unit, $\overline{x}$ is the mean of all values across all *n* units, and *w*_ij_ is the spatial weight matrix that is a function of the distance that describes the neighborhood of spatial units. A positive Moran’s I indicates the existence of clusters of similar values, while a negative Moran’s I indicates clusters of dissimilar values. Moran’s I closer to 0 indicates weak autocorrelation [52].

## Results and Discussion

### Deprivation index and its OWA scenarios

**Figure 3:**
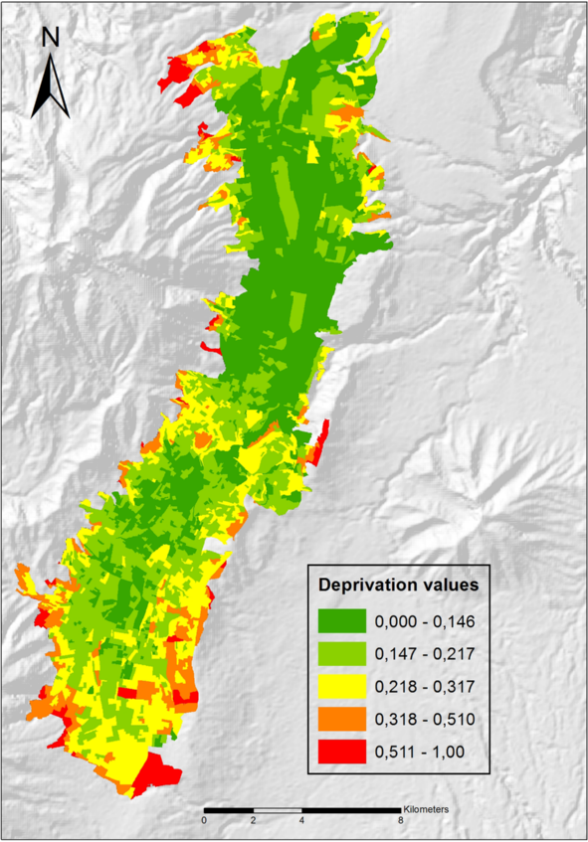
**AHP**-**based deprivation index result.**

The AHP-based deprivation Index results (Figure 3) display the presence of medium and high levels of deprivation (0,511 to 1,000) in specific zones of the study area, even though most of Quito City has low values of deprivation. Higher levels of deprivation appear at the edges of the study area, and represent relatively recently settled neighborhoods created by socio-economically more deprived people. On the other hand, lower deprivation levels (0,000 to 0,146) are commonly present on the northern side of the City, a part of Quito generally inhabited by people with better socio-economic conditions. Moderately deprived areas are located in the south, a very industrial and commercial area, traditionally inhabited by blue collar workers. These results coincide with what was explained in the study area description and confirm the consistency (Consistency ratio CR: 0,0019) of the AHP weights derived from the experts’ judgments. The AHP-based deprivation Index has been shown to be very useful to evaluating levels of socio-economic deprivation considering our human rights-based approach: deprivation caused by unsatisfied needs due to a lack of basic services and capabilities related to human rights. For example, people with lower levels of education and health that live in unworthy households with limited or no access to basic services are considered to have high levels of deprivation in many socio-economic dimensions.

**Figure 4:**
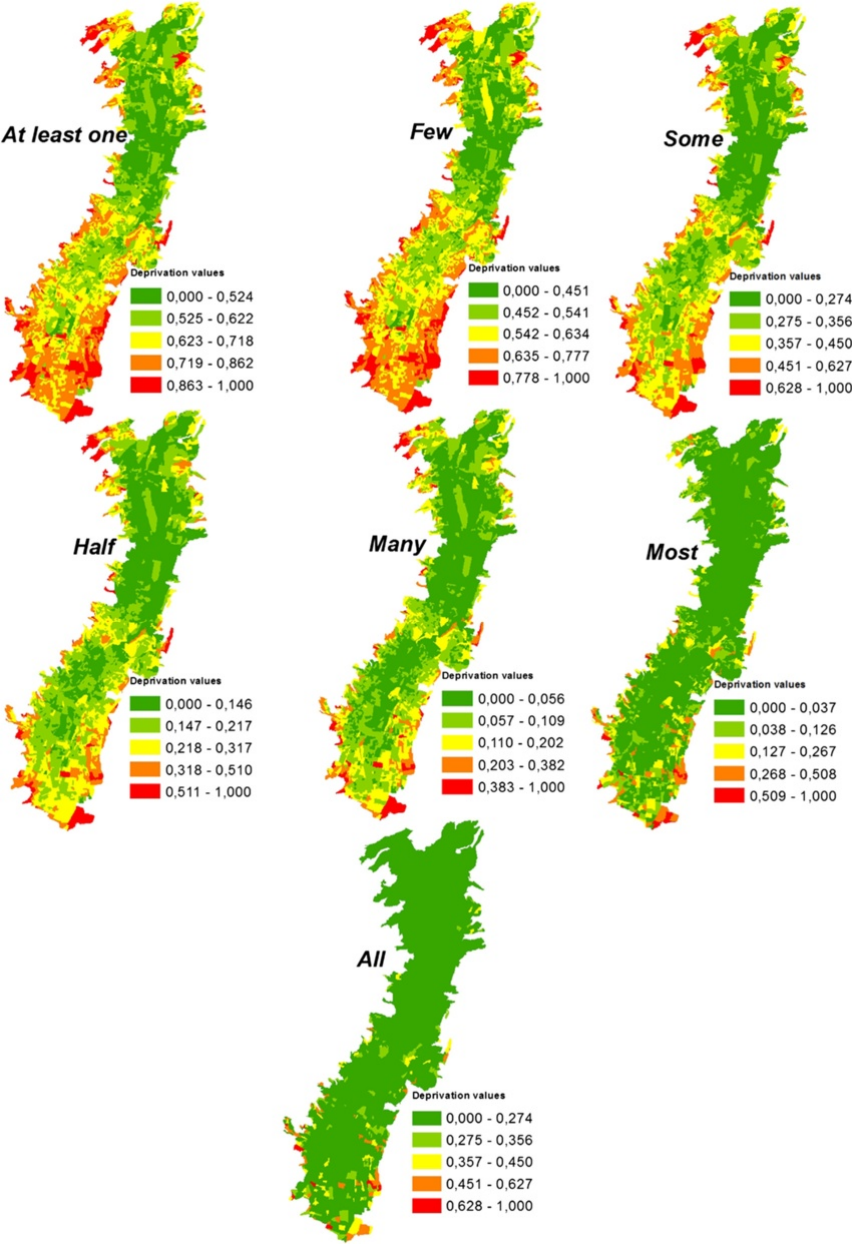
**OWA scenarios of the deprivation index.**

Seven OWA scenarios were obtained: “At least one”, “Few”, “Some”, “Half”, “Many”, “Most” and “All” (Figure 4). The “At least one” deprivation scenario represents the extremely optimistic strategy, where the highest possible deprivation values are shown for each census block. In this scenario, decision makers can have high risk-taking propensity to weigh more highly “positive outcomes” [53], with “positive outcomes” meaning “higher values of deprivation criteria”. In this scenario, the indicator with the maximum value gets full weighting [54]. The results are census blocks with higher deprivation scores than the AHP-based deprivation index. The “All” deprivation scenario represents the extremely pessimistic strategy where the lowest possible deprivation values are shown for each census block, which means that the indicator with the minimum value gets full weighting [54] and the census blocks have lower deprivation scores than the AHP-based deprivation index. The “Half” deprivation scenario is the equivalent to the AHP-based deprivation index, because the equal order weights are applied for all indicators. The deprivation scenarios “Few” and “Some” are relatively optimistic scenarios where greater order weights are assigned to higher criterion values and smaller weights are assigned to the lower criterion values. The deprivation scenarios “Many” and “Most” are relatively pessimistic scenarios where greater order weights are assigned to lower criterion values and smaller weights are assigned to the higher criterion values.

The deprivation scenario with the linguistic quantifier “All” (logic AND) is considered the “worst-case scenario” [44] and in the case of our study, the worst-case scenario means that no action needs to be taken regarding the socio-economic deprivation in almost the entire Quito City territory. Nevertheless, this scenario could be useful to detect the most deprived areas, and can discern areas where taking immediate action is required to reduce socio-economic deprivation. On the other hand, the deprivation scenario with the linguistic quantifier “At least one” (logic OR), representing extreme optimism, shows larger deprivation areas. With this strategy, a larger number of deprivation areas should be considered for socio-economic recuperation, but this may not be feasible for decision makers due to time- and financial constraints. The “Half” scenario means that if the decision makers’ risk-taking is neutral, only the AHP deprivation index constructed based on the experts’ judgments can be considered. The “Few” and “Some” scenarios could support decision making that identifies areas where an extensive social-improvement program could work for most of the city, while the scenarios “Many” and “Most” could support decision making that focuses on taking action in highly socio-economically deprived areas without excessive financial/time investment.

### Spatial relationships between the different deprivation measures and health factors

The GWR models show the goodness of fit results for the spatial relationships between the different deprivation measures and health factors (Table 6). A Gaussian kernel was used to solve each local regression and the extension of the kernel is determined using the Akaike Information Criterion (AIC). The AIC is a relative measure of a statistical model quality that takes into account the statistical goodness of fit and the tradeoff of the parameters used in the model. There is no range of values for this measure and the best model is considered to be the one with the lowest AIC value. The GWR models that have the best goodness of fit are the “AHP-based” model and the “Half” model. Other models with low AIC values are the “Some” and “Many” models, showing the importance of using OWA scenarios as tradeoffs between a neutral scenario and extreme scenarios when describing deprivation and health interactions. The models mentioned, “AHP-based”, “Half”, “Some” and “Many”, are also models that represent similar proportions of the dependent variable variance: between 58% and 59%. However, this does not mean that these regressions produce an optimal dependent variable prediction in all locations.

**Table 6:** **GWR Statistics for all regressions performed**

	**AHP**-**based**	**At least one**	**Few**	**Some**	**Half**	**Many**	**Most**	**All**
**AIC**	18470,02	18743,45	18634,98	18483,08	18470,02	18505,63	18684,81	19653,30
**R** ^**2**^	0,59	0,50	0,53	0,58	0,59	0,59	0,57	0,36

Each regression is identified in the table with the explanatory variable of deprivation.

Moran’s I statistics identified clusters in the residual values of all GWR performed (Table 7). Clustering with high levels of significance indicate that explanatory variables are missing. In Moran’s I the null hypothesis is the random distribution of values. Table 7 shows a random distribution in the models that showed the best goodness of fit (“AHP-based”, “Half”, “Some” and “Many”) as well as in the “Most” model. This means that these deprivation scenarios, in combination with the ‘distance to health services’ factor, could be explanatory variables to predict the percentage of people that have never had a live birth. The models with the presence of residual clusters with high levels of significance (“At least one”, “Few”, “All”) are models that do not completely explain the health dependent variable.

**Table 7:** **Moran**’**s I statistics for the residuals of all regression performed**

	**AHP**-**based**	**At least one**	**Few**	**Some**	**Half**	**Many**	**Most**	**All**
**Moran**’**s Index**	−0,005	0,051	0,035	−0,000	−0,006	−0,008	0,006	0,215
**z**-**score**	−1,083 (Random)	10,198 (Clustered)	7,130 (Clustered)	−0,049 (Random)	−1,076 (Random)	−1,478 (Random)	1,175 (Random)	42,908 (Clustered)
**p**-**value**	0,279	0,000	0,000	0,961	0,282	0,139	0,239	0,0000

Each regression is identified in the table with the explanatory variable of deprivation.

## Conclusion

Our AHP-based deprivation index is a multidimensional index that considers a rights-based conceptual approach useful to representing deprivation in dimensions of education, health, employment and housing. We conclude that our deprivation index has the potential to explain the socio-economic deprivation in the study area accurately because i) the important rights-based indicators used, ii) the consistency of experts’ opinions in the AHP method, and because iii) the several alternative deprivation scenarios allow decision makers to identify urgent zones that can be addressed efficiently and also to the identification of a broader spectrum of zones that can be addressed using more resources. These OWA deprivation scenarios can be considered useful tools for decision makers and health planners. The different decision strategies offer different options when dealing with socio-economic deprivation in the study area. If decision makers decide not to use the AHP-based deprivation index, they can opt for a variety of tradeoff deprivation scenarios (“Few”, “Some”, “Many” and “Most”) that can guide them to where their work will yield better results by saving time and financial resources. The “All” scenario is also interesting when it comes to identifying very deprived zones. These zones represent bigger gaps in the quality of life, and people living there should be considered a priority by health planners and city authorities. The GWR models show that the deprivation index and its scenarios can be related to health factors, and that several deprivation scenarios in combination with the ‘distance to health services’ factor, could be considered explanatories variables to predict the percentage of people that have never had a live birth.

One limitation of this study is that no analysis of uncertainty was elaborated for the OWA scenarios. Even though this is not an objective of this article, we consider that a future study can incorporate uncertainty analysis for different OWA deprivation scenarios. Another limitation is that this study does not develop a complete statistical deprivation-health factor model. We reiterate that the GWR and Moran’s I analyses should only be seen as an exploratory analysis, and more research regarding this issue is needed. Future research could include the incorporation of more explanatory health variables that could interact with the AHP-based deprivation index and the OWA deprivation scenarios. The identification of additional health problems that can be explained to some degree by the methods implemented in this study is also important. Further work can include variations of the Multi-Criteria evaluation used, for example, the use of different techniques to obtain criteria weights and order weights for the deprivation indicators.

This study has several strengths, and can be considered as one of the first instances where Multi-Criteria evaluation methods such as AHP and OWA are utilized to create a deprivation index and deprivation scenarios. A strength of this study is that the AHP-OWA approach captures quantitative and qualitative information to produce different scenarios that are useful for decision makers when faced with different decision strategies due to constraints in time and financial resources. A further value of our study is that the OWA method is spatial in the sense that it aggregates the criteria for each census block depending on their values, and this aggregation is done for all linguistic quantifiers. Another strength of this work is the fact that the OWA procedure was automated with the development of the Python toolbox, which allows more efficient calculation of OWA deprivation scenarios for future studies.

The methodology described in this study can be applied in other regions of the world to develop spatial deprivation indices based on Multi-Criteria analysis.

An important contribution of this study is that the mixed method of applying AHP to calculate deprivation criteria weights and OWA to create different deprivation scenarios is a methodology that can be carried out in other studies beyond Latin America. The indicators considered in this study are common Population and Housing Census variables. However, as AHP and OWA methods are techniques that can be adapted to specific problems and phenomena, future studies can use the methodology presented here considering different deprivation indicators. Furthermore, the methods and results showed in this study can be considered as important tools to support health planners and decision makers.
